# Optimizing Extraction Methods for Bioactive Polysaccharides from *Rosa rugosa* and *Rosa damascena*

**DOI:** 10.3390/foods14183211

**Published:** 2025-09-15

**Authors:** Sawaira Ashraf, Muhammad Zahid Ashraf, Baohe Miao, Xinxin Zhao

**Affiliations:** 1 Institute of Urban Agriculture, Chinese Academy of Agricultural Sciences, Chengdu National Agricultural Science & Technology Center, Chengdu 610213, China; sawairaashraf717@gmail.com; 2 State Key Laboratory for Conservation and Utilization of Subtropical Agro-Bioresources, South China Agricultural University, Guangzhou 510006, China; ashrafzahid848@gmail.com

**Keywords:** *Rose* species, bioactive compounds, polysaccharide extraction, extraction techniques, antioxidant activity, industrial applications

## Abstract

*Rosa damascena* and *Rosa rugosa*, which are the two most commercial species in the *Rosa* genus, are used to make rose oil, cosmetics, and functional foods. The majority of polysaccharide constituents of both species is structurally diverse and demonstrates promising biological activities, such as moisturizing, immunomodulation, and antioxidant activity. The extraction technique has a significant impact on the yield, purity, and bioactivity of polysaccharides. Traditional extraction methods (hot water, ethanol) are simple and economical, yet they typically produce low yields and degrade sensitive compounds. Novel extraction methods (pressurized liquid extraction, enzyme-assisted extraction, ultrasound-assisted extraction, microwave-assisted extraction, supercritical fluid extraction) offer higher efficiency, selectivity, and sustainability, while better preserving polysaccharide structure and bioactivity. This review serves as a comparative summary of conventional versus novel extraction methodologies of polysaccharides from *R. damascena* and *R. rugosa*, with particular consideration towards the yield, polysaccharide structural integrity, sustainability, and industrial conduct of each methodology. In addition, it summarizes the distribution and functional role of selected polysaccharides in the various organs of the plants, while also providing an overview of their antioxidant mechanisms and potential bioactive applications in health. Challenges and critical factors that surround specific species, standards for processes, and extraction methods, and that therefore appeal to time and economic considerations, are identified. In efforts to optimize the extraction methodology, the high economic and functional potential of the *Rosa* species can be maximized in the interest of healthy, functional consumables for the pharmaceutical, nutraceutical, and cosmetic industries.

## 1. Introduction

The genus Rosa, belonging to the Rosaceae family, encompasses more than 100 species and several hybrids cultivated worldwide for their ornamental, aromatic, and medicinal value [1]. Among these, *Rosa rugosa* and *Rosa damascena* are especially significant industrially and are widely utilized in the production of rose oil, cosmetics, herbal medicines, and functional foods [2]. *Rosa damascena* is an essential part of the rose oil and cosmetics industries and is grown mostly in Bulgaria, Turkey, Iran, India, and various parts of the Middle East [3]. In contrast, *Rosa rugosa* is widely grown in China, Japan, Korea, and parts of Europe, particularly in coastal areas due to its strong adaptability to saline and sandy soils [4]. Both species have a wide range of uses in cosmetics, functional foods, herbal medicine, and aromatherapy because they are abundant in essential oils (particularly citronellol, geraniol, and phenylethyl alcohol), phenolic compounds, flavonoids, vitamin C, and organic acids in addition to polysaccharides [5]. Rose hips, notably rich in vitamin C, have been investigated for their anti-inflammatory effects and show potential to alleviate joint discomfort, particularly in osteoarthritis [6]. Additionally, rose water, derived from petals, is prized in cosmetic formulations for its moisturizing and anti-inflammatory properties, while traditional herbal applications of rose petals include digestive and calming benefits [7]. The essential oils of *Rosa damascena* are also known for their influence on the limbic system, contributing to mood enhancement and anxiety reduction, which underpins their frequent use in aromatherapy [8].

Beyond essential phenolic compounds and oils, recent agricultural and pharmacological research has increasingly focused on the bioactive polysaccharides present in various parts of rose plants, including leaves, stems, petals, and fruit residues [9,10]. These polysaccharides, which are complex carbohydrates composed of repeating monosaccharide units linked by glycosidic bonds, exhibit significant biological activities such as moisturizing, immunomodulatory, and antioxidant effects, and they also contribute to plant structural integrity [11]. The structural features of these macromolecules, including sugar composition, molecular weight, and branching patterns, critically influence their biofunctional properties.

Polysaccharides from the *Rosa* species have been very potent in their antioxidant capacity. Antioxidants play a direct role in reducing oxidative stress; they accomplish this by chelating the metal ions which act as pro-oxidants and scavenging free radicals [12]. The methods of extraction have great influence on how effective these antioxidant activities can be because, through different techniques, the purity, yield, and also preservation of the structure of the polysaccharides are achieved [7]. Conventional extraction is still practiced because it is simple and cheap; however, it has certain drawbacks in terms of low yield and partial degradation of the components. Among the more advanced methods are microwave-assisted, enzyme-assisted, pressurized liquid, ultrasound-assisted, and supercritical fluid extraction, which are more efficient, environmentally friendly, and selective [13].

This review mainly concentrates on *Rosa rugosa* and *Rosa damascena* to give a detailed summary of the polysaccharide makeup in different parts of the plant, critically assess old and new ways of extraction, and compare what affects structural wholeness and how well extraction works. By focusing on these two industrially important types, this work gives a specific view that connects practical uses with basic study in medicine, food industry, and beauty products. This review also emphasizes the sustainability dimension of extraction methods, their potential for industrial application, and opportunities for the valorization of by-products within the circular bioeconomy framework.

## 2. Polysaccharides in Different Parts of Rose Plant

**Figure 1:**
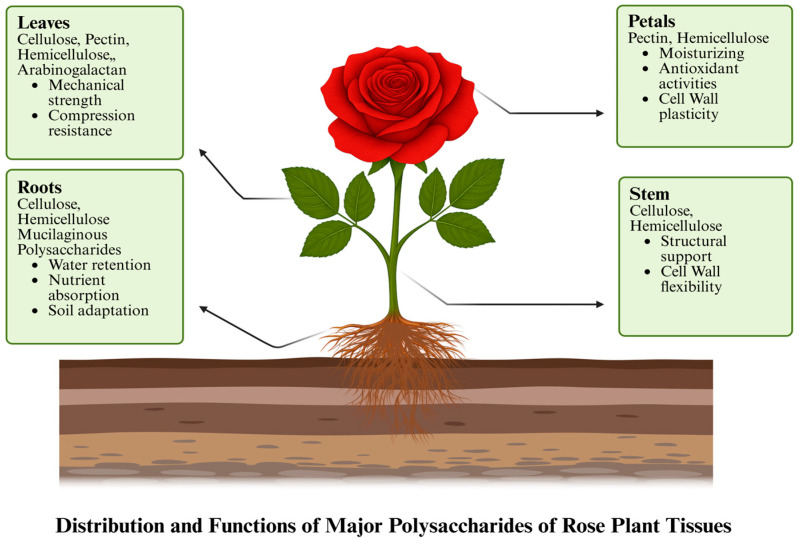
This schematic diagram illustrates the spatial distribution of key polysaccharides and their associated physiological functions in the leaves, petals, stems, and roots of *Rosa rugosa* and *Rosa damascena*.

Polysaccharides are important bioactive substances that are present in the leaves, petals, roots, and stem of rose plants (*Rosa* spp.). Comprising extended chains of monosaccharide units, these complex carbohydrates perform a variety of roles in the plant, including defense against environmental stresses, energy storage, and structural support. The spatial distribution of major polysaccharides in rose organs is illustrated in Figure 1.

### 2.1. Polysaccharide Composition

Rose plants (*Rosa* spp.) constitute a rich source of structurally diverse polysaccharides that are distributed across multiple organs, including leaves, petals, stems, and roots [14]. The main classes of these plant-derived polysaccharides are pectin, cellulose, hemicelluloses—the xylans and mannans—lignin, glucans, and arabinogalactan [15,16]. They play very critical roles in how the plant can adapt to environmental stresses and also in the structure and metabolism of the plant.

#### 2.1.1. Leaves

Rose leaves comprise a varied polysaccharide composition. This includes pectin, cellulose hemicelluloses, and arabinogalactan [16]. Pectin forms the major constituent of the middle lamella, mediates intercellular connections, and elasticizes to the cell wall; it also contributes to defense responses and to controls of water retention [17]. Cellulose forms the major structural component of the mechanical strength imparted by the cell wall and rigidity by cellulose [18]. Arabinogalactan is related to cell wall proteinaceous elements responsible for cell wall signaling and extensibility [19]. Together, these polysaccharides provide flexibility, resilience to environmental stress, as well as structural stability.

#### 2.1.2. Petals

Rose petals contain an abundance of hemicelluloses and pectin, which play important roles in cell wall integrity and development [20]. Hemicelluloses, arabinans, and xylans further give strength to cell wall hydration and flexibility [21]. From these bioactive polysaccharides, cosmetics and medicines are derived from rose petals, particularly anti-inflammatory creams and moisturizers [22]. Pectin is otherwise galacturonic acid present in cell walls, hydrating the walls and giving them plasticity. It also lends free radical scavenging antioxidant activity [23].

#### 2.1.3. Stems

Rose stems have high levels of cellulose and hemicelluloses, particularly xylans and xyloglucans, which give them flexibility as a vital structural component [16,24]. Cellulose forms sturdy microfibrils that add to the mechanical strength of the stem, making it able to stand against environmental pressures [25]. Hemicelluloses make a binding link with cellulose to take on more flexible and coherent cell walls while adding to the support for nutrient and water transport within the plant [26]. Lignin content is also higher in stems; therefore, they are not easily degradable and also offer strength [27].

#### 2.1.4. Roots

**Figure 2:**
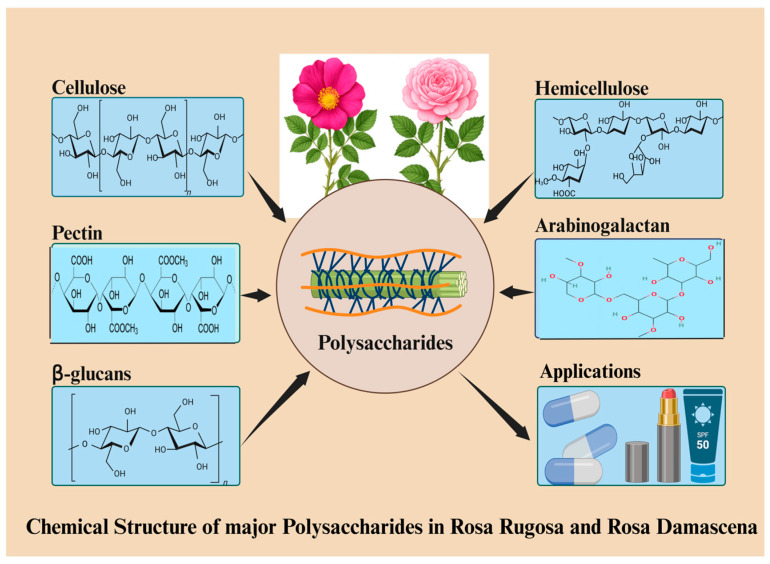
This diagram illustrates the chemical structures of the major polysaccharides identified in *Rosa rugosa* and *Rosa damascena*, including cellulose, pectin, hemicellulose, arabinogalactan, and β-glucans. The central panel emphasizes the collective polysaccharide composition of rose tissues, while surrounding boxes depict the structural diversity of individual polysaccharides. These structures underpin the physicochemical properties and biological activities of rose-derived polysaccharides, which are linked to applications in medicine, food, and cosmetics.

The set of polysaccharides found in rose roots is different from other type and comprises cellulose, hemicelluloses, aerial parts, pectin, and quite large amounts of mucilaginous polysaccharides like arabinogalactan and glucans [28]. Cellulose gives strength and structure to soil compaction; pectin and hemicelluloses help roots grow in compact soil and regulate cell wall flexibility [26]. Mucilaginous polysaccharides help nutrient absorption, help water retention, and also help with soil microbiota; this shows the adaptation of roots to subterranean environments [29]. However, for industrial applications, harvesting roots means destroying the entire plant, which raises sustainability issues. Therefore, rather than large-scale valorization, root-derived polysaccharides are more pertinent to the scholarly characterization of the complete polysaccharide profile of *Rosa* species [30]. Polysaccharides from petals, leaves, and even spent stems or by-products of the rose oil industry are more feasible sources for realistic and sustainable biorefinery processes because they permit continuous or post-harvest recovery without endangering plant survival. The chemical structures of the major polysaccharides present in *Rosa rugosa* and *Rosa damascena* are shown in Figure 2.

Though the literature presents a detailed account of the compounds that make up the polysaccharide content of a variety of organs of rose plants, it is important to appreciate the fact that the composition is dynamic and influenced by several biological and environmental factors, among others. As an example, the high pectin content in petals contributes to maintaining cell wall flexibility and hydration of the cell wall, which is vital to releasing aromas and softening of the petals [31]. The difference between the polysaccharide profiles distinguished in various studies suggests, however, that polysaccharide structure and yield are highly dependent on growth conditions, plant species, and harvest date [32]. This deviation complicates the standardization of extraction processes and does not facilitate the achievement of consistent bioactivity in industrial uses [33]. The development of the standardized methods of compositional profiling and the investigation of the influence of environmental stress factors on polysaccharide synthesis are, therefore, the primary direction of work in the future. The discoveries will enable more narrow extraction methods that will produce a high yield and activities that have specific applications in food, cosmetics, and pharmaceuticals. Furthermore, Table 1 shows a summary of the relative abundance and distribution of the main polysaccharides in various organs of rose plants. The petals are high in pectin and hemicelluloses; the leaves and stems are predominated by cellulose and hemicelluloses; and the roots have other mucilaginous polysaccharides that facilitate their own special physiological needs. Stems and roots have a considerable amount of lignin, which makes them structurally firm. Such diversification of composition of polysaccharides provides the specific biological roles of each organ in the growth, development, and adaptation of the plant to the environment.

**Table 1:** Distribution, relative abundance, and functional roles of major polysaccharides in different parts of the rose plant.

Polysaccharide	Petals	Leaves	Stems	Roots	Functional Roles	References
Pectin	High (>10%)	Moderate (5–10%)	Low (1–5%)	Low (1–5%)	Hydration, Cell wall plasticity, Antioxidant properties	[20,22,34,35]
Lignin	Very Low (<1%)	Low (1–5%)	High (>10%)	High (>10%)	Protection against degradation, Structural rigidity	[36]
Cellulose	Low (1–5%)	Moderate (5–10%)	High (>10%)	High (>10%)	Structural support, Mechanical strength	[36,37]
Hemicelluloses	Moderate (5–10%)	High (>10%)	High (>10%)	Moderate (5–10%)	Cell wall cohesion, Nutrient transport, Flexibility	[20,37]
Arabinogalactan	Moderate (5–10%)	Moderate (5–10%)	Low (1–5%)	Moderate (5–10%)	Signaling, Water retention, Cell wall extensibility	[38,39]
Glucans (incl. β-glucans)	Moderate (5–10%)	Low (1–5%)	Low (1–5%)	Moderate (5–10%)	Immunomodulatory effects, Antioxidant, Water retention	[39,40]
Mucilaginous polysaccharides	Low (1–5%)	Low (1–5%)	Low (1–5%)	High (>10%)	Nutrient absorption, Water retention, Soil adaptation	[38,39]
Xyloglucans/ Xylans	Low (1–5%)	Low (1–5%)	High (>10%)	Moderate (5–10%)	Cell wall flexibility especially in roots and stems	[37]
Galacturonic acid-rich pectin	High (>10%)	Moderate (5–10%)	Low (1–5%)	Low (1–5%)	Antioxidant properties, Major component in petals and fruit	[34]
β-glucans	Moderate (5–10%)	Low (1–5%)	Low (1–5%)	Moderate (5–10%)	Anti-inflammatory, Antioxidant, Immunomodulatory activities	[40]

### 2.2. Antioxidant Properties

Polysaccharides extracted from *Rosa damascena* and *Rosa rugosa* have garnered considerable interest due to their pronounced antioxidant activities, which play a vital role in both plant defense and potential human health applications [41]. These antioxidant effects are primarily attributed to several interrelated mechanisms, which are described below.

#### 2.2.1. Free Radical Scavenging

Rose polysaccharides are effective in neutralizing reactive oxygen species (ROS) such as superoxide anions and hydroxyl radicals [42]. This is mainly due to hydroxyl groups and characteristic sugar residues in their structures, which can donate electrons or hydrogen atoms to stabilize the free radicals and to break the oxidative chain reactions [43]. Recent studies have demonstrated that rose-derived polysaccharides possess strong free radical scavenging ability. In 2,2-diphenyl-1-picrylhydrazyl (DPPH) assays, for instance, *Rosa rugosa* polysaccharides demonstrated up to 68% 2,2-diphenyl-1-picrylhydrazyl (DPPH) radical scavenging at 2 mg/mL, whereas *Rosa damascena* extracts obtained an IC_50_ of 1.2 mg/mL [44]. These results confirm that rose polysaccharides are effective hydrogen and electron donors, stabilizing free radicals in vitro.

#### 2.2.2. Metal Ion Chelation

The process of chelation of transition metal ions, especially Fe^2+^ and Cu^2+^, is another worthwhile antioxidant mechanism [45]. Rose polysaccharides sequester those pro-oxidant metals, limiting their involvement in Fenton-like reactions and resulting in the reduced production of highly reactive hydroxyl radicals and the decrease of the oxidative stress [46]. Quantitative evaluations have shown that rose polysaccharides effectively bind transition metals [47]. For example, *Rosa damascena* root extracts demonstrated chelation efficiencies exceeding 40% at 1.5 mg/mL, whereas *Rosa rugosa* polysaccharides decreased Fe^2+^ availability by almost 55% at 2 mg/mL [33]. This ability to sequester pro-oxidant metals highlights their role in preventing Fenton-type radical formation.

#### 2.2.3. Enhancement of Endogenous Antioxidant Enzymes

The polysaccharides that are derived of roses have been indicated to have the capacity to elevate the activity of endogenous antioxidative enzymes, including catalase and super oxide dismutase [48]. It also increases the defense mechanism of the plant due to this enzymatic strengthening and aids redox homeostasis in surviving the stresses [49]. Their antioxidant activity strongly correlates with their structure to include molecular weight, monosaccharide composition, uronic acid contents, or branching pattern [49]. Such elements affect their interaction with ROS and metal ions, their bioavailability, and their functional work in biological environments [50]. In vivo assays further support these findings. In animal models under oxidative stress, the administration of rose polysaccharide fractions from *Rosa rugosa* fruit markedly increased catalase (CAT) activity by 28% and superoxide dismutase (SOD) activity by 35% [51]. Similar outcomes were seen with extracts from *Rosa damascena*, where polysaccharide supplementation improved redox homeostasis by increasing glutathione peroxidase (GPx) activity [52].

In order to substantiate the practical usefulness of rose polysaccharides, there is an urgent need to examine the correlation between in vitro antioxidant tests and in vivo as well as food systems models. Although there is an encouraging report of antioxidant activities in rose polysaccharides, a major problem is the inter-assay variability of antioxidant capacities, which is largely due to assay variation in extraction procedure, sample preparation, and testing conditions. Additionally, the relationship between polysaccharide structure (e.g., molecular weight, branching, and sugar composition) and antioxidant efficacy remains poorly understood; hence, there is little chance of predicting the effect of extraction conditions on the maintenance of antioxidant activity. When these problems are dealt with by means of combined structural–function experiments and standardized antioxidative assays, the full promises of rose polysaccharides as endogenous antioxidants in foods, cosmetics, and health products will be achieved. Moreover, antioxidant activity must be translated into industrial use, which means that any extraction process must have the property of structural integrity and must be scalable and environmentally sustainable. All these antioxidant activities aid not only the in-house protection and lifespan of rose plant, but they also indicate the possibility of rose polysaccharides as food preservatives, cosmetics, and herbal medicine active ingredients. They are desirable for the production of health-promoting products, and because of their multi-functional antioxidant capabilities, they are also likely to prove useful in dealing with oxidative damage in many industrial contexts.

## 3. Extraction Methods

**Figure 3:**
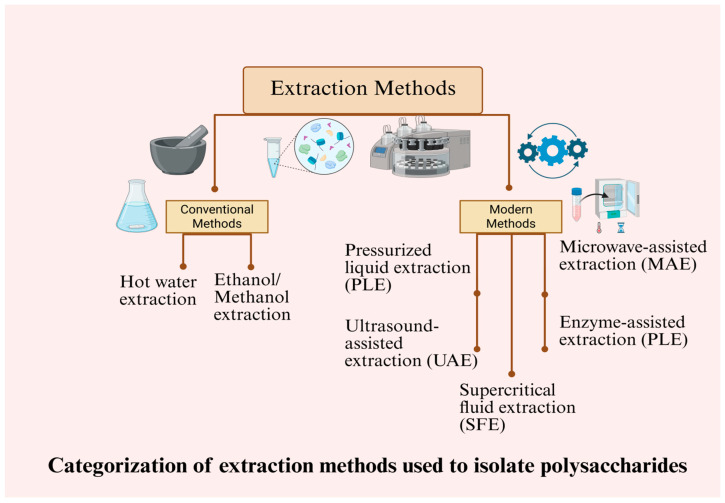
Categorization of extraction methods used to isolate polysaccharides in the rose plant (*Rosa* spp.). Modern methods of extraction, i.e., pressurized liquid extraction (PLE), ultrasound-assisted extraction (UAE), microwave-assisted extraction, (MAE), enzyme-assisted extraction (EAE), and supercritical fluid extraction (SFE), have a higher extraction efficiency and selectivity, include more sustainable methods, and are particularly useful.

A diverse amount of the traditional and modern ways can be used to extract polysaccharides from different parts of rose plants with the aim to isolate these bioactive components, leaving them structurally and functionally complete. The following is a description of the conventional and modern methods of extracting polysaccharides in rose plants. The extraction methods applied to isolate polysaccharides from rose plants can be categorized into several groups, as shown in Figure 3.

### 3.1. Conventional Methods

Polysaccharides are extracted through traditional methods such as hot water extraction and extraction by ethanol or methanol using sections of rose plants. Due to their affordability, ease of use, and simplicity, these techniques have been used for several decades.

#### 3.1.1. Hot Water Extraction

Hot water extraction is one of the most common conventional methods of separating the polysaccharides of rose plants mainly in the petals, leaves, stems, and roots. To liquefy such water-soluble polysaccharides such as pectin, arabinogalactan, and glucans, plant material is heated or boiled within distilled water [53]. As a rule, the plant material undergoes degradation with the temperature range from 60 °C to 100 °C, depending upon the focus of the targeted polysaccharide [54]. Extraction will remove liquids and leave solids, which are then filtered out, and typically, the polysaccharides are found by either precipitating (with alcohol, such as ethanol) or drying (evaporating the water) [55].

Hot water extraction is widely used because it is environmentally friendly, affordable, and simple since no toxic organic solvents are generated in the process [56]. The method is especially individual in the industry of pharmaceuticals, nutraceuticals, and cosmetics since it preserves the bioactivity of polysaccharides [13]. This process can be easily scaled-up to industrial uses, and the aqueous processing conditions are well-suited to avoid the risk of bioactive materials going bad [57].

There are severe demerits of hot water extraction despite the advantages that it has. A relatively high temperature and long periods of extraction time that could sometimes run long into hours may result in the partial hydrolysis of polysaccharides that reduces molecular weight, altering their functional properties [58]. This is not a very efficient method to eliminate certain poorly soluble-in-water polysaccharides or those that are strongly bound to other components of the cell wall, such as proteins and lignin [59]. Proteins and phenolics could be co-extracted alongside other contaminants, making the purifying processes even tougher [60]. To get rid of its disadvantages, it is often utilized in conjunction with more advanced methods or perfect extraction conditions as a means to enhance the level of yield and purity. Although hot water extraction is straightforward and inexpensive, it necessitates high temperatures and lengthy processing times, which can raise energy consumption and possibly break down delicate compounds [61]. Its simplicity makes it widely applicable at the industrial scale, but optimization is required to lower energy consumption. Following extraction, residual biomass can be further valorized for composting or animal feed.

#### 3.1.2. Ethanol/Methanol Extraction

The separation of polysaccharides using ethanol and methanol extraction methods in polysaccharide purification is common when polysaccharides of higher molecular weight are prepared [57]. These subsequent, after initial aqueous extraction, alcohol-based procedures typically serve as a type of precipitation step in separating the polysaccharides from other contaminants that may have co-extracted into the mix, including proteins, phenolics, and low-molecular-weight molecules [62]. The extraction after aqueous extraction includes adding ethanol or methanol residually to the solution containing polysaccharides in a concentration of about 70–90% (*v*/*v*) [32]. Due to the destruction of hydration shells around the molecules of polysaccharides and modifications in the polarity of the solvent, the low solubility of polysaccharides in high alcohol concentration favors their precipitation [63]. Precipitate is then collected by centrifugation or filtration, and further cleaning is performed to make the precipitate purer [64]. Relative pure compounds, with structural integrity, are obtained when ethanol or methanol is used to precipitate high-molecular-weight polysaccharides selectively [65]. The method is well established in the synthesis of polysaccharides to be used in pharmaceutical and nutraceutical applications, as it is easy scale and can be adjusted to downstream processes [66]. Additionally, the end product will have minimal chemical residues since ethanol and methanol are gaseous and can be removed very easily. Although there are advantages to conducting ethanol or methanol precipitation, the procedure has disadvantages. This method finds wide application in the production of polysaccharides with pharmaceutical and nutraceutical uses due to its easiness, scalability, and compatibility with down streaming processing [66]. Moreover, the final product will contain minimal compounds of chemicals since ethanol and methanol are volatile and can be easily removed.

Although ethanol or methanol precipitation is effective, it also has limitations. This method is uneconomical and less eco-friendly since it often requires very large quantities of alcohol [67]. Polysaccharides of low molecular weight with strongly alcohol-soluble or alcohol–water mixtures are more difficult to obtain using this technique. Other purification techniques that can be applied to enhance purity and selectively remove low-molecular-weight fractions to overcome the limitation of ethanol or methanol precipitation in the extraction of the complex sugar or polysaccharide of rose plant parts are membrane filtration, enzyme procedures, and chromatography [59]. Several traditional techniques have been employed for extracting polysaccharides from different parts of rose plants, as summarized in Table 2.

### 3.2. Modern Extraction Methods

**Figure 4:**
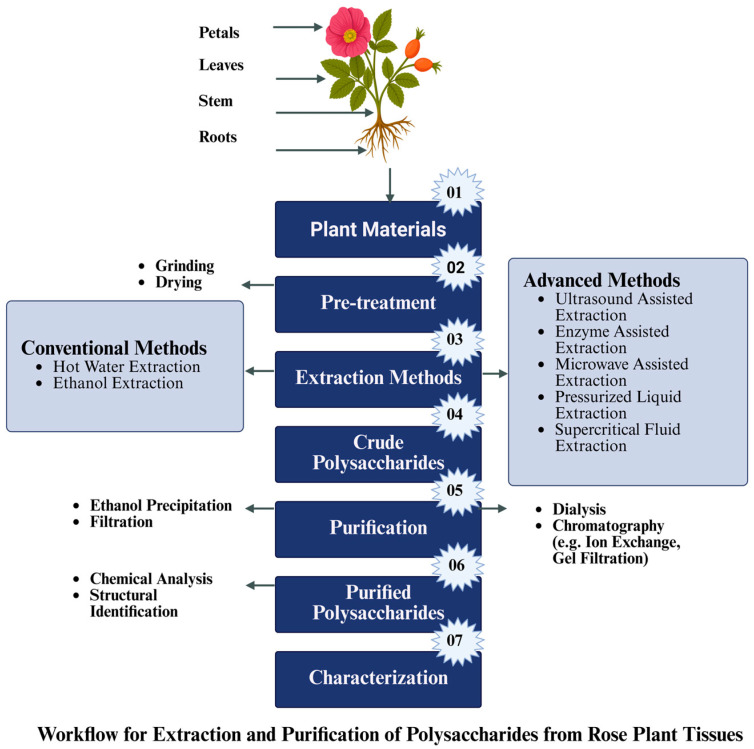
This figure is the representative diagram used to explain the systematic protocol applied in the extraction, purification, and characterization of polysaccharides obtained using different body parts of the rose plant such as petals, leaves, stems, and roots. The stage starts by gathering plant materials (Step 1) and pre-treatment, which includes grinding and drying (Step 2). During Step 3, extraction techniques are used; they are divided into traditional (hot water, ethanol, etc.) and modern (ultrasound-assisted, enzyme-assisted, microwave-assisted, pressurized liquid, and super-critical fluid extraction). The obtained crude polysaccharides (Step 4) are followed by purification steps (Step 5) such as ethanol precipitation, filtration, dialysis, and chromatography (e.g., ion exchange, gel filtration) to obtain a purified product. Purified polysaccharides (Step 6) thus obtained are analyzed using chemical and structural analyses (Step 7) so as to characterize them completely. The given workflow offers an exhaustive scheme to obtain functional active polysaccharides of rose plant tissues with a view of its use in pharmaceutical, nutraceutical, and cosmetic industries.

When compared to traditional methods, modern extraction practices have more efficiency and better bioactivity preservation that significantly promote polysaccharides isolation in rose plant constituents. Modern techniques like pressurized liquid extraction (PLE), enzyme-assisted extraction (EAE), microwave-assisted extraction (MAE), ultrasound-assisted extraction (UAE), and supercritical fluid extraction (SFE) are considered to be totally in line with the needs of a proper separation of polysaccharides among the components of rose plants. The general workflow for extraction and purification of rose polysaccharides from plant tissues is illustrated in Figure 4.

#### 3.2.1. Pressurized Liquid Extraction (PLE)

The trending and efficient method used in the recovery of polysaccharides in rose plant parts is pressurized liquid extraction (PLE), which possesses several advantages relative to traditional methodologies. To enhance the solubility and diffusion of polysaccharide components of plant matrices, PLE employs solvents under increased temperatures and pressure, typically ranging between 100 and 150 °C [77]. This process is faster, and the efficiency of extraction is improved, as a result of the high pressure that reduces the vaporization point of the solvent; thus, the extraction occurs at higher temperatures than the standard boiling points [78]. This method is also particularly effective in breaking down the complex cellular compounds in plant tissues, and bioactive polysaccharides can be released with the minimum of degradation [79]. Among the key positive potential applications of PLE, it can take to the extraction of polysaccharides quicker than more traditional methods, i.e., hot water extraction, and with a lower energy requirement [80,81]. By using PLE, extraction conditions such as temperature, pressure, and solvent can be better controlled to extract specific fractions of polysaccharides [82]. Due to this tailoring, it is now possible to extract high-value bioactive polysaccharides in rose plants, selectively enhancing their functional properties to be used in the food, cosmetic, and pharmaceutical industries [81]. PLE has high yields and preserves the bioactiveness of polysaccharides; thus, it could be considered an appropriate way to extract polysaccharides in an efficient and sustainable manner.

#### 3.2.2. Ultrasound-Assisted Extraction (UAE)

The application of ultrasound-assisted extraction (UAE) has proven to gain various bioactive chemicals out of the other parts of the rose plant [83]. In a study conducted by Um et al., it was revealed that an increase in UAE parameters was able to enhance the extraction of ascorbic acid and phenolic and flavonoid components of *Rosa rugosa* fruit [84]. The best parameters were identified as 50% ethanol concentration, 50 °C temperature of extraction, and 40 min of extraction time. In this situation, the yield of total phenolic content (TPC) and total flavonoid content (TFC) was maximized, and the extracts exhibited good antioxidant activity [84]. Similarly, investigations on *Rosa damascena* have revealed the possibility that UAE can isolate helpful polysaccharides with antioxidant properties [85]. It is suggested that UAE could be useful because it has been successfully applied in the extraction of polysaccharides in related species, in which *Rosa roxburghii*, is one; thus, we could also apply it in the process of obtaining polysaccharides of *Rosa damascena* [86]. To illustrate, in some conditions, Chen and Kan managed to obtain yields of 6.59% and 1.34%; when it comes to extracting polysaccharides in *Rosa roxburghii* fruit, UAE conditions have been optimized [87]. The isolated polysaccharides possessed significant antioxidant activities, with an in vitro ability to scavenge hydroxyl, superoxide, and DPPH radicals. Based on these findings, UAE could be one of the forms of polysaccharide extraction in *Rosa damascena* rich in antioxidants, and it deserves further investigation [87]. Some major advantages of applying UAE to rose plants are high yields and better bioactivity of the extracted chemicals [88]. This technique uses effects of cavitation, where the walls of plant cells are destroyed, to enhance the mass transfer and make it easier to release intracellular contents [89]. Although ultrasound-assisted extraction increases yield and shortens extraction times, its high electricity consumption may have a significant impact on sustainability. Adoption in industry is feasible, particularly with the use of renewable energy. Following extraction, solid residues can be transformed into fiber additives or biochar, promoting a circular bioeconomy.

#### 3.2.3. Microwave-Assisted Extraction (MAE)

For extracting polysaccharides high in antioxidants, there is a widely used extraction technique called microwave-assisted extraction (MAE) to extract them from various parts of the rose plant. A study on the fruit of *Rosa roxburghii* determined the optimal parameters of a microwave-aided enzyme extract procedure using the response surface approach [69]. Polysaccharide yield was 36.21% (±0.62), with the set of ideal parameters being a microwave power of 575 W, an enzyme dose of 6.5 g/mL, a liquid-to-material ratio of 13.5:1 mL/g, and extraction time of 18 min [69]. One of the recent improvements in MAE is the active use of deep eutectic solvents (DES) to increase extraction yield [90]. In a recent study, a microwave-assisted DES strategy was utilized for the extraction of aromatic chemicals and cellulose using rose petals. By controlling the composition of the DES, the researchers were able to obtain a removal rate of 40.80% of the lignin and an anthocyanin extraction yield of 173.71 mg/g [90]. Marked advantages of utilizing MAE with rose plants involve elevated activity in antioxidants, accelerated extraction rate, and improved yields of polysaccharides [91]. This method involves efficient energy transfer and rapid heating so that plant cell walls can be ruptured so that their intracellular contents can be liberated and improve mass transport [92]. Microwave-assisted extraction is an effective method because it enables quick extraction with less solvent usage. Large-scale use, however, might be restricted by specialized equipment and high power requirements. Its industrial potential is growing, especially in areas that target high-value compounds. Spent materials can be utilized as ingredients for dietary fiber or for the recovery of biomass energy. MAE preserves the functional properties of bioactive chemicals and, at the same time, leads to higher extraction efficiency; thus, it can be adopted as a viable technique to obtain high-quality extracts of rose plants that are abundant in antioxidants. According to recent research, employing deep eutectic solvents (DES) in conjunction with microwave heating greatly increases extraction yield and preserves antioxidants while using fewer dangerous organic solvents [93]. Because of its non-toxic and biodegradable components, DES is becoming more and more acknowledged as a green solvent for the extraction of polysaccharides, supporting the goals of sustainable processing.

#### 3.2.4. Enzyme-Assisted Extraction (EAE)

Enzyme-assisted extraction (EAE) is one of the widely-known methods that are used to extract bioactive polysaccharides out of plant materials, especially rose plants [94]. Freeing intracellular polysaccharides using EAE boosts the extraction by a process involving the hydrolysis of cell wall compounds by certain enzymes [95]. As an example, the extraction of the residues of *Rosa rugosa* was studied, and the extraction was optimized using a joint cellulase and pectinase enzyme. With optimal conditions, namely a 1:15 g:mL material to liquid ratio, an addition of 1.9% of enzyme, a 1:1 ratio of cellulase to pectinase, an enzymatic hydrolysis temperature of 47 °C, and a time of 84.5 min, the yield of polysaccharide was 4.308 percent ± 0.03% [96]. On the same note, a mixture of a 2:1 cellulase-to-pectinase ratio was used to enhance the EAE process of *Rosa roxburghii* pomace to yield 4.79% polysaccharides at 60 °C, a pH of 4.0, and 2.5% cellulase concentration [97]. EAE, especially when used on rose plants with enzymes such as pectinase, enhances the recovery of polysaccharide contents in rose plant tissues by efficiently dissolving the wall content components [33]. Under mild conditions, enzyme-assisted extraction increases yield while requiring less energy than traditional heating. Enzyme recovery and cost, however, present difficulties for industrial use [98]. The method is scalable, particularly in the pharmaceutical and food industries where enzyme technologies are well-established. For instance, by-products can be valued as residues rich in bioactive compounds or fractions rich in proteins.

#### 3.2.5. Supercritical Fluid Extraction (SFE)

Supercritical fluid extraction (SFE) is becoming an extremely popular and environmentally friendly way of removing bioactive components of plants, including ones like the polysaccharides present in rose plants [99]. The process employs the use of supercritical carbon dioxide (SC-CO_2_), which exhibits both gas properties of diffusivity as well as liquid properties of solubility. This enables a better yield of extraction and more efficient penetration of the plant matrix [100]. Important advantages of SFE compared to conventional technologies is low temperatures of extraction, a reduction in the amount of solvent used, and the opportunity to extract specific target substances after determining temperature and pressure values [101]. The functional biomolecules possessing the antioxidant properties can be most adequately obtained by subjecting the rose plant polysaccharides to SC-CO_2_, and this ensures that there is minimal degradation of the thermolabile components and that it will preserve excellent purity and bioactivity [102].

In case of SFE applications, the extraction of rose plants exemplifies SFE as having high efficiency and efficiency in separating bioactive compounds of a plant, in this case, petals, leaves, and fruits [103]. Since SC-CO_2_ and polar polysaccharides do not mix, the non-polar nature of SC-CO_2_ typically restricts its ability to directly extract polar polysaccharides; thus, the yield and solubility of polysaccharide can generally be significantly enhanced by co-solvents such as ethanol or water [104]. The high fitness activity is possible to extract polysaccharides with and present them with their structure and functional properties with the help of SFE after optimizing operations factors, such as pressure, temperature, and the proportion of co-solvents [105]. SFE involves less toxicity and potentially less solvent residue due to the fact that it uses a cleaner method when compared to traditional solvent-based extractions, which makes it a more viable extraction method when it comes to use in food, medicine, and cosmetics [61]. Supercritical CO_2_ extraction is environmentally friendly because it is very selective and stays away from hazardous solvents. However, the requirement for CO_2_ recycling systems and high-pressure equipment raises expenses and energy consumption, which might restrict the use of low-value products. It works best in cosmetic or high-value nutraceutical applications. Remaining fat can be further processed to make animal feed or dietary fiber.

The extraction of the polysaccharides in roses with the SFE method has additional potential, especially for businesses that will need the high-purity, bioactive constituents in health-promoting products. Rose-derived polysaccharides are becoming more popular in nutraceuticals, functional foods, and cosmetic formulations due to their potent immune–modulatory and antioxidant properties [105]. SFE’s capacity to create solvent-free extracts is in line with the growing need in the pharmaceutical and cosmetics sectors for sustainable and natural components.

#### 3.2.6. Integrated and Hybrid Extraction Approaches

Synergistic effects can be achieved by combining two or more techniques, which lowers energy and solvent consumption while increasing extraction efficiency, selectivity, and sustainability [106]. Enzyme-assisted ultrasound extraction (EAE + UAE), for example, has demonstrated that ultrasonic cavitation breaks down plant cell walls, increasing enzyme penetration and speeding up hydrolysis [107]. This leads to higher yields with lower enzyme dosages and shorter processing times [107]. Similar to this, microwave-assisted deep eutectic solvent systems (MAE + DES) combine green solvents with quick microwave heating to greatly increase the solubility and antioxidant retention of polysaccharides while using fewer harmful solvents [93]. When compared to either technique alone, ultrasound-assisted DES extraction (UAE + DES) has also shown enhanced polysaccharide yields and antioxidant qualities [59]. Because they support the objectives of sustainable processing and the frameworks of the circular bioeconomy, such hybrid strategies are being used more and more in food, nutraceutical, and biowaste valorization systems [108]. Future research into these synergistic combinations could enable scalable, eco-friendly industrial applications of rose-derived polysaccharides. A comparison of different extraction methods for rose polysaccharides is presented in Table 3.

### 3.3. Factors Affecting Extraction Efficiency

Extraction of polysaccharides in *Rosa damascena* and *Rosa rugosa* depends on a mixture of processing conditions with the properties of the plant material. The optimization of these factors must also be meticulous to enhance the yield, maintain the structural integrity, and promote the functionality of the extracted polysaccharides. The following are the most critical ones.

#### 3.3.1. Extraction Temperature

One of the important parameters that influence the solubility and diffusion of polysaccharides in plant tissues is temperature. An increase in extraction temperature generally leads to higher yields due to enhanced solvent penetration and disruption of cell walls [57]. As an example, hot water extraction is usually carried out at the temperature range of 60 °C to 100 °C in order to extract water-soluble polysaccharides, including pectin and arabinogalactan [111,115]. Nevertheless, harsh temperatures or long heating times may cause some partial hydrolysis and degradation to the polysaccharides, lowering their molecular weight and bioactivity [116]. Hence, a favorable temperature interval should be determined to achieve the equilibrium between extraction efficiency and structure activity of polysaccharides.

The optimization of extraction temperature is crucial not only for maximizing yield but also for preserving the structural integrity and bioactivity of rose polysaccharides. Although high temperatures may be useful in disrupting cell walls, it may also achieve the severe degradation of thermolabile polysaccharide fractions, making them less useful. The application of moderate extraction temperatures is usually desirable for applications in the food and nutraceutical sectors, since it has been shown to simultaneously provide extraction performance, retain the bioactive quality, and yield compliance with the regulatory demands.

#### 3.3.2. Extraction Time

The duration of extraction has significant effects on the extent of polysaccharide recovery. Time of extraction can be longer, facilitating more solubilization and diffusion and, subsequently, the increased yield [32]. Nevertheless, prolonged extraction may also favor the destruction of polysaccharides and employment of other compounds with impurities, including proteins and phenolics that might complicate post-extraction purification and, in addition, reduce bioactivity [83]. The results of response surface methodology have demonstrated that extraction time has a moderate range with optimal enzyme concentration, and its range gives a good match of yield versus quality [117].

To obtain the successful extraction of polysaccharides and minimize the possibility of degradation, as well as the co-extraction of other non-desirable compounds, the extraction time should be closely monitored. To determine the optimal minimum required time of extraction, real-time monitoring and kinetic modeling should be used in industrial practice that will contribute to increased efficiency of the process and the stability of the product quality. Longer extraction reactions can improve the yield, or they can degrade product purity and functional activity.

#### 3.3.3. Liquid to Solid Ratio

The ratio of plant material to solvent is very important in order to ensure that an adequate concentration gradient exists to drive the mass transfer of the polysaccharides into the solvent. Increasing liquid-to-solid ratios usually increases the high capacity of extraction, which is due to more solvent accessibility and diffusion coefficient [117]. Large volumes of a solvent may dilute the extraction, add unnecessary expenses to the processing, and may make downstream concentration difficult. The ideal ratios of the rose polysaccharide extraction usually range between 20 and 40 mL/g besides the method of extraction used [83].

It is recommended to optimize the liquid-to-solid ratio during large-scale operations to achieve a balance between extraction performance, solvent economy, and environmental footprint, thereby ensuring cost-effective and sustainable production. Although an increase in the ratios may aid mass transfer and better yields, this will also lead to the use of more solvent and an overall increment in the cost of process.

#### 3.3.4. Enzyme Concentration and Type

In enzyme-assisted extraction (EAE), the kind and concentration of the enzyme (cellulase, pectinase, and hemicelluloses) are consequential to the efficient breakdown of cell boundary and extraction of a partial polysaccharide [83]. The general tendency when increasing enzyme dosage is increased yield because the polysaccharides are released by breaking down the cell wall. Still, the use of excessive amounts of enzymes can result in over-degradation of the polysaccharides or a leftover contamination of the enzyme over-representation, and this must be optimized [118].

It is important to validate the use of enzymes at the pilot scale and to carry out a cost–benefit analysis at the industrial level, such that the use of enzymes is technically and economically viable in the case of food-grade ingredient manufacturing. EAE has significant potential in the selective and efficient recovery of polysaccharides in conditions that are relatively mild though the enzyme concentration and selection would need to be optimized to avoid over-degrading target polymers and further avoided in the final product mixed with the enzyme.

#### 3.3.5. Ultrasonic Power and Duration

Ultrasound-assisted extraction (UAE) utilizes ultrasonic waves to disrupt plant cell membranes, allowing the solvent to penetrate directly into plant cells. Thus, the challenge is controlling the power of ultrasound and the extraction duration, extracting as many polysaccharides as possible but ensuring that thermal or mechanical degradation does not occur [33]. For example, discontinuous ultrasound at a power of 360 W, an extraction time of 25 min, and at 60 °C provided optimal yield and purity of polysaccharides from *Rosa roxburghii* [119]. These studies point to significant benefits of polysaccharide extraction through ultrasound-assisted extraction for yield and, possibly, process intensification. On the contrary, ultrasound-assisted extraction is very dependent on the power of the ultrasonic strength and duration of the extraction process. If ultrasonic power is too strong, or time too long, then sensitive polysaccharide aggregates can become thermally or mechanically degraded, lowering bioactivity. Therefore, in order to synergize polysaccharide extraction efficiency and functional integrity, the discontinuous or pulsed ultrasound concept must be explored in parallel with quality testing of the extract in real time.

#### 3.3.6. Solvent Type and Concentration

Water is the most commonly used solvent for extracting hydrophilic polysaccharides, owing to its safety and practical applicability. Alcohols (e.g., ethanol or methanol) are mainly employed for protein and phenolic purification and precipitation because they ultimately lessen the solubility of polysaccharides, which aids in separating these molecules from proteins and phenolics [120,121]. The concentration of alcohol (70–90%) can be optimized for polysaccharide precipitation efficiency and purity; however, for food and nutraceutical use, water and ethanol are typically the best solvents because of their safety profile and existing regulations; nevertheless, investigating additional green solvents may allow for better extraction selectivity and sustainability for the extraction of rose polysaccharides. Solvent type and concentration directly influence downstream processing, safety, and extraction selectivity.

#### 3.3.7. Plant Material Characteristics

The type, provenance, and plant part of the rose (*Rosa* spp.) used as raw material will likely have marked effects on extraction yields and polysaccharide composition. Variations could stem from inherent differences in the plant-produced cell wall structure, polysaccharide content, and related extractives that may act as confounding variables [116,117]. From a standard extraction point of view, pre-extraction steps like drying, grinding, and defatting would likely affect extraction efficiency from the perspective of increasing surface area and improving solubility or solvent availability. To strategize favorable polysaccharide extraction from *Rosa damascena* and *Rosa rugosa*, a detailed understanding of the dictating factors above and their interactions at work is critical. Polysaccharide extraction may serve better under less-extractive conditions when potentially pooling knowledge from combined modern extraction methods, such as using a multi-step extraction approach using enhanced extraction methods and enzyme-assisted extraction paired with ultrasound extraction [96]. The optimization of extraction conditions, e.g., response surface methodology (RSM) to systemically modulate extraction parameters, could improve reproducibility and scalability for use in industries [117].

Using Life Cycle Assessment (LCA) metrics to measure environmental impacts like energy use, solvent consumption, and waste generation is another way to assess extraction efficiency from a sustainability standpoint [122]. Utilizing green chemistry concepts (such as safer solvents, waste reduction, and energy efficiency) and process intensification techniques (such as combining enzyme-assisted extraction with ultrasound-assisted extraction) can minimize processing time, solvent consumption, and environmental impact while preserving polysaccharide bioactivity [123,124].

Plant material variability of species, organs, or pre-extraction treatments likely poses one of the most significant barriers to standardization and reproducibility in extraction procedures. The development of rapid and non-destructive methods to quantify and process raw material inputs provides a pursuit for increased process consistency and better predictably in final extraction outcomes in an industry context. To achieve the efficient, sustainable, and scalable production of bioactive polysaccharides from roses, it is crucial to grasp extraction parameters and optimize them in a systematic way. Combining green processing principles, real-time process monitoring, and thorough characterization of processing materials should facilitate the mission of making research laboratory innovations become commercial processes, supporting high-quality functional ingredients for the modern food industry. The comparative performance of major extraction methods for rose polysaccharides is summarized in Table 4.

## 4. Challenges in Extraction and Optimization

### 4.1. Challenges

Numerous scientific and technical obstacles must be overcome in order to extract antioxidant-rich polysaccharides from different regions of the rose plant. Complex plant cell wall architectures, chemical compositional diversity, and polysaccharide sensitivity to extraction conditions are the causes of these difficulties. A thorough analysis of the main obstacles to extraction process optimization may be found below.

#### 4.1.1. Yield Limitations

Obtaining polysaccharides from rose plants presents a considerable challenge because of poor yield, especially from the woody parts, such as the stems and roots [129]. The woody parts of the plant contain relatively high levels of cellulose, hemicelluloses, and lignin, which act together to provide a rigid structure and stabilize the polysaccharides. This is unlike the softer cell structures present in the leaves and petals [130]. Moreover, woody surfaces are often too tough to effectively disrupt with conventional hot-water extraction processes, and yields are rarely satisfactory [131]. Acid-assisted extraction or enzymatic hydrolysis can improve solubilization rates; however, overly harsh treatment may cause hydrolysis, lowering polysaccharide molecular weight and compromising bioactivity [60,132,133]. Therefore, one of the key challenges associated with enhancing yield is developing and optimizing pre-treatment methods while maintaining the structural integrity of the polysaccharides of interest. Furthermore, because it requires uprooting the plant, using roots as a raw material presents both ecological and financial issues. Renewable biomass (petals, leaves, and by-product residues) should be given priority in future extraction operations because they are plentiful and work well with sustainable supply chains.

#### 4.1.2. Degradation During Extraction

The chemical and thermal instability of polysaccharides makes them difficult to extract. A large number of bioactive polysaccharides are highly sensitive to temperature fluctuations and concomitant or prolonged exposure to strong solvents, especially those containing antioxidant activity [10]. Chemical degradation due to high temperature leads to the depolymerization of polysaccharides, resulting in reduced molecular weight and decreased antioxidant activity of the polysaccharides. High temperatures are often required to enhance solubility and yield [22]. Prolonged extraction times significantly increase the likelihood for an oxidatively deteriorative event to occur, which can severely compromise the usefulness of the polysaccharides [134]. Innovative methods such as ultrasound-assisted extraction (UAE) and microwave-assisted extraction (MAE) can be utilized to enhance extraction efficiency and reduce thermal degradation [135]. These alternate methods maintain the bioactivity of polysaccharides due to increased control over the application of temperature and length of extraction time.

#### 4.1.3. Variation Between Rose Species

The optimization of extraction methods is complicated by the variability in polysaccharide composition across different rose species. Polysaccharide structural qualities and antioxidant activity differ due to genetic variation among the *Rosa* species, which complicates the effort from a single extraction methodology [136]. Additionally, environmental factors—often related to soil type and climate or changing with the seasons—might impact the biochemical components of rose plants, which can result in consequences on antioxidant activity and yield [137]. Different plant parts may have polysaccharide contents that considerably vary, even within the same species; hence, extraction should follow a species- and tissue-specific approach [138]. To ensure consistency with polysaccharide extractions, standardization is needed when choosing the plant materials, placing them in conditions to be collected, and when choosing the pre-extraction processing method (e.g., cuttings, if vegetative plant parts are being harvested for polysaccharide extraction) [32,69]. The significant genetic variability and biochemical variability not only between species but also physiological plant parts and environmental conditions shows that we do need to optimize extraction protocols that are species and tissue-specific. The variations between the *Rosa* species have potential for the discovery of new polysaccharide structures and bioactivities, but they simultaneously present obvious challenges with such protocols for standardization or bulk bioprocessing. To guarantee the reproducibility and scalability of extraction processes, the standardization of raw materials, including pre-treatment steps, is essential in addition to species and plant part variations. The final yield and bioactivity of polysaccharides are influenced by pre-extraction processes like drying, grinding, and enzymatic pre-treatment, which must be tailored for each species and plant part [139].

In commercial applications where batch-to-batch uniformity is crucial, reproducibility and consistency in polysaccharide yield and functional properties may be compromised if plant material selection, harvesting, and pre-extraction processing are not carefully standardized. Therefore, it is crucial that future research prioritizes the development of effective quality control techniques and uniform sourcing procedures. Furthermore, the incorporation of sophisticated analytical methods for quick raw material characterization may make it easier to determine the best extraction conditions for particular *Rosa* species and tissues, thereby enhancing the dependability and economic feasibility of polysaccharide products derived from roses. The main challenges in extracting rose polysaccharides, along with corresponding optimization strategies, are summarized in Table 5.

### 4.2. Optimization Strategies

#### 4.2.1. Enhancing Extraction Efficiency

Numerous factors such as temperature, time, enzyme concentration, and solvent composition are significant in influencing extraction efficiencies [141]. The main way to improve extraction efficiencies is through the use of the correct solvents. Although they are eco-friendly, aqueous extraction efficiencies may not be sufficient for extracting some bioactive polysaccharides [142]. Ethanol/methanol-based extractions, or deep eutectic solvent (DES) extractions, can improve the solubility of specific polysaccharide fractions while maintaining bioactivity [143]. Substitutes for organic solvents are eco-friendly solvents with biodegradable and non-toxic ingredients found in DES [144].

#### 4.2.2. Sustainable and Green Extraction Techniques

Extraction methods that are sustainable and green in nature are generating interest as the approaches, while increasing extraction efficiency, have decreased adverse effects on the environment [145]. The approaches of ultrasound-assisted extraction (UAE) and microwave-assisted extraction (MAE) both aid in disrupting cell walls, decreasing extraction time, and reducing solvent use [135]. A premier way to perform extraction in a safe and pure manner is through a solvent-free method using supercritical fluid extraction (SFE) using CO_2_ [101]. Finally, subcritical water extraction (SWE) represents a distinctive technique that employs hot pressurized water for polysaccharide isolation, eliminating the need for organic solvents [146]. Nonetheless, these innovative methods have high extraction efficacy and are environmentally acceptable. Utilizing a statistical approach known as response surface methodology (RSM) allows for the systematic optimization of extraction conditions [147]. This approach provides simulations of multi-factor interactions including temperature, time, and solvent ratio for optimally effective extraction conditions while conserving resources [148]. Furthermore, by lowering dependency on petrochemical solvents and promoting adherence to green chemistry frameworks, the use of DES and subcritical water extraction strengthens sustainability even more [149]. These green extraction techniques strike a balance between environmental responsibility and efficiency when used in conjunction with statistical optimization tools like RSM.

#### 4.2.3. Pre-Treatment Methods for Improved Extraction

When extracting polysaccharides from rose plant materials, pre-treatment enhances accessibility. Mechanical disruption (i.e., grinding, ultrafine milling) is a strategy adopted to enhance solvent access via an increased surface area [61]. Cell wall disruption can also be achieved through alkaline treatments or mild acid hydrolysis, enabling higher extraction yields while preserving polysaccharide integrity [140]. Pre-treatment strategies, solvent selection, tuning of parameters, and the development of sustainable extraction techniques are all incorporated into a holistic approach for maximizing yields of antioxidant-rich polysaccharides from rose plant materials. Using statistical optimization techniques and advanced extraction options (enzyme-assisted and/or green extraction) will allow researchers to maximize the yield while minimizing negative effects on the environment. Future work should focus on scaling up efficient methods for usable industrial size while being affordable and sustainable.

## 5. Applications and Future Perspectives of Rose Polysaccharides

### 5.1. Current Utilization of Rose Polysaccharides

**Figure 5:**
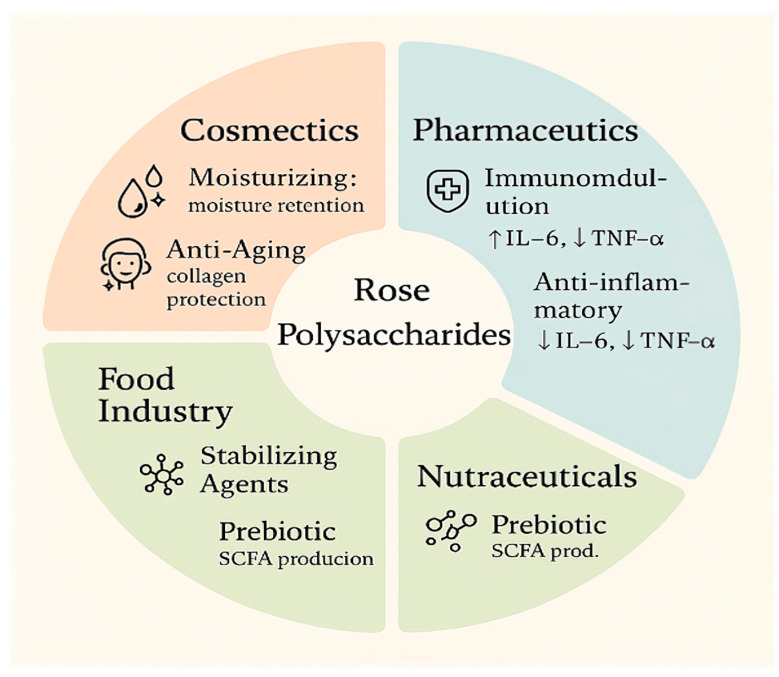
This figure depicts the following predominant fields of applications and biological functions of rose polysaccharides: moisturizing and anti-aging in cosmetic science, immunomodulatory and anti-inflammatory and other purposes in pharmaceutical science, prebiotic function in nutraceuticals, and stabilizing agents and prebiotics in food technology. Abbreviations: SCFA, short-chain fatty acids; IL-6, interleukin-6; TNF-α, tumor necrosis factor-alpha.

Polysaccharides produced from *Rosa damascena* and *Rosa rugosa* are now exploited across many industries due to their unique bioactivities and functional characteristics. The diverse applications and biological functions of rose polysaccharides in cosmetics, pharmaceuticals, nutraceuticals, and food technology are summarized in Figure 5.

#### 5.1.1. Cosmetic Industry

Due to their ability to mitigate oxidative stress and promote skin health, polysaccharides obtained from the petals and hips of rose plants are increasingly used in skincare products [150]. For example, *Rosa rugosa* petal polysaccharides have added to anti-aging serums at concentrations of 1–5% to improve skin hydration and minimize fine lines [2]. They are used at a concentration from 0.5 to 3% in moisturizing creams to increase the elasticity of the skin [2]. Furthermore, polysaccharides act as natural humectants by creating a moisture-retaining barrier on the dermal surface, which improves skin hydration [151]. For example, *Rosa rugosa* petal-derived polysaccharide fractions exhibit strong anti-free radical activity, which may diminish UV-induced skin damage while simultaneously reducing visible signs of aging [67]. Thanks to their anti-inflammatory properties, polysaccharides from roses are also excellent ingredients for sensitive or acne-prone formulations; they calm irritated skin and reduce redness [152].

Rose-derived polysaccharides have been reported to exhibit topical anti-aging effects by inhibiting collagen degradation and promoting stabilization of the extracellular matrix [153]. Studies on by-products of *Rosa damascena* demonstrate that pectic polysaccharides recovered from rose oil waste share rheological properties with commercially available citrus pectin and could be used in gel formulations to develop serums and masks [2]. Furthermore, the high concentration of vitamin C in rose petals contributes with polysaccharides to an increase in antioxidant activity, which may provide protection from environmental pollutants and improve skin softness [154]. These properties enhance the use of rose-derived polysaccharides as multifunctional ingredients in moisturizers, toners, and anti-aging serums catering to consumer preference for sustainable/environmental/natural sources in cosmetics [155].

#### 5.1.2. Pharmaceuticals

The bioactive capacity of rose polysaccharides for anti-inflammatory, antidiabetic, and anti-cancer purposes makes them potential pharmaceuticals [10]. Rich fractions in polysaccharides from *Rosa rugosa* hips and petals possess indirect anticancer activity by modulating the immune response and direct anticancer effects by inducing apoptosis in lung and colon cancer cell lines [91], and they may potentially be used therapeutically for chronic inflammatory diseases like arthritis or other pro-inflammatory conditions due to their ability to inhibit pro-inflammatory enzymes such as COX-1 and COX-2 [116]. Polysaccharides from seedless chestnut rose (*Rosa sterilis*) fruits show strong α-glycosidase inhibitory activity to help manage glucose levels in the blood following meals [91]; thus, they act as viable nutraceuticals or functional foods for the treatment of diabetes. Likewise, ascorbic acid and phenolic compounds were found to be contributing factors to the potential hepatoprotective and renal-protective characteristics of rose hip polysaccharides, as shown in preclinical research [154]. The capacity of rose polysaccharides to manage oxidative stress pathways also supports the utilization of these potential functional foods or nutraceuticals for formulations directed at preventing cardiovascular or neurological diseases [5]. *Rosa rugosa* polysaccharides have been shown to have the ability to lower inflammatory markers when added to capsule formulations at doses of 100–500 mg daily [40].

#### 5.1.3. Nutraceuticals

Polysaccharides derived from roses show promising potential in the nutraceutical industry, where they can be used as prebiotics, dietary supplements, and functional food ingredients [2]. Because of their potent antioxidant and immunomodulatory properties, they can be included in products that promote health and prevent conditions linked to oxidative stress [2,156]. *Rosa sterilis* fruit polysaccharides, for instance, have demonstrated strong α-glycosidase inhibitory activity, indicating potential for controlling blood sugar and managing diabetes [157]. In a similar vein, polysaccharides isolated from the hips of *Rosa rugosa* show strong anti-inflammatory and antioxidant properties that could promote gastrointestinal and cardiovascular health [40]. Furthermore, their function as prebiotic agents is highlighted by their capacity to alter gut microbiota and encourage the synthesis of advantageous metabolites like short-chain fatty acids [158]. These properties indicate that rose polysaccharides can serve as valuable nutraceutical ingredients, supporting both disease prevention and overall wellness.

#### 5.1.4. Food Industry

There is an increasing trend in the food sector for the use of rose polysaccharides as advantageous adjuncts and naturally derived preservatives. Utilizing the fruit of oil-bearing roses (*Rosa damascena*), which provides polyphenolic and polysaccharide-rich extracts, ensures that perishables have an extended shelf life by combing oxidation mechanisms and microbiological growth [33]. For example, pectic polysaccharides harvested from rose oil by-products replace artificial thickeners and create an essential, stable gel medium in food products, like jams and jellies, and dairy applications with the right levels and ratios of calcium ions present in the food product [159]. To obtain the best texture and consistency, 0.5–2% of *Rosa damascena* pectin can be added to jams as a gelling agent [160]. In addition to preserving foods and beverages, rose polysaccharides enhance nutritional benefits. Polysaccharides from *Rosa sterilis* fruit exhibited significant antioxidant activity in linoleic acid systems [32], exceeding traditional food additives to reduce oxidative rancidity. Furthermore, bodily biochemistry tests of rose petals revealed comparable amounts of ascorbic acid and related photochemicals as those found in soft fruits like strawberries. Therefore, edible roses provide an alternative source of vitamin C and dietary fiber [161]. Thus, these functional properties can be developed into supplements to fortify snacks or beverages in a manner that also promotes digestive and immune health.

### 5.2. Future Utilization Perspectives

The extraction and application of rose polysaccharides have achieved notable progress; yet, there remain vast opportunities to further enhance their utilization through innovative technologies, expanded species exploration, and industrial-scale development. Advancing these areas will not only improve extraction efficiency and product functionality but will also enable sustainable and economically viable production.

#### 5.2.1. Emerging Technologies

The recovery of antioxidant-rich polysaccharides from rose plants may undergo a revolution with the use of extraction technologies. Under ideal conditions (enzyme concentration: 2.5%, pH 4.0, 60 °C), enzyme-assisted extraction, especially with cellulase and pectinase combinations, has demonstrated potential in increasing polysaccharide yields from *Rosa roxburghii* pomace, attaining a 4.79% yield while maintaining bioactivity [155]. The efficiency of extracting polysaccharides from *Rosa roxburghii* fruits was also enhanced by intermittent ultrasound-assisted enzymatic extraction; at 60 °C and a 40 mL/g liquid–solid ratio, the optimal parameters produced 15.03% crude polysaccharides [125]. Deep eutectic solvents (DESs), which are emerging green solvents, provide sustainable alternatives. In Bletilla striata, DESs based on choline chloride urea enhanced polysaccharide yields by 36.77% when compared to traditional methods, and they also had higher antioxidant activity [67,162]. Future studies could adapt DESs for rose polysaccharide extraction, limiting solvent toxicity and energy utilization [40,116]. Nanotechnology-based innovations, such as nano-encapsulation, could further enhance the stability of polysaccharides and enable their targeted delivery in pharmaceuticals or cosmetics; however, applications to rose-derived compounds remain largely underexplored [163,164]. It is expected that the efficiency and sustainability of rose polysaccharide recovery will be greatly increased by the use of technologies such as enzyme-assisted, ultrasound-assisted, and green solvent-based techniques. To guarantee that gains in laboratory yield and bioactivity are sustained in commercial production settings, successful industrial implementation will necessitate thorough safety evaluations, scalability validation, and strict process parameter optimization.

#### 5.2.2. Rose Species Exploration

Present research concentrates on a small number of species, such as *Rosa roxburghii* and *R. rugosa*, but there is still a great deal of genetic variation among more than 150 *Rosa* species. For instance, the hips of *R. rugosa* have a glucan content of up to 12.26 g/100 g, while the petals and leaves exhibit unique polysaccharide profiles that have anti-inflammatory and anticancer properties [40]. Wild species and underutilized cultivars, such as Schwarze Madonna (331.95 mg cyanidin-3-glucoside/100 g fresh weight) or *R. sterilis* (strong α-glycosidase inhibitory activity), may provide novel polysaccharide variations with distinct bioactivities [2,165]. Linking structural variation to functional qualities and mapping the distribution of polysaccharides require comparative studies across species and plant parts (such as petals, hips, and leaves) [166]. Breeding programs for industrial uses could be accelerated by the identification of genetic markers for high-yielding cultivars using Omics technologies (genomics, Metabolomics) [167,168]. In order to identify high-yielding and bioactive genotypes, systematic comparative studies and the use of Omics technologies will be crucial. Research efforts should be expanded to include a wider range of *Rosa* species, including wild and underutilized cultivars, as this is likely to uncover novel polysaccharide structures with distinct functional properties. This will support targeted breeding and resource selection strategies for industrial applications.

#### 5.2.3. Industrial Scaling

The following key obstacles must be overcome in order to move from lab-scale optimization to industrial production:

##### Process Standardization

Although temperature (60–81 °C) and liquid–solid ratios (21–40 mL/g) are optimized via response surface methodology (RSM), scaling these conditions requires energy-efficient reactors and real-time monitoring systems to ensure consistency [147]. Standardized protocols and real-time monitoring systems must be developed as extraction moves from laboratory to industrial scale in order to guarantee process reproducibility and product consistency. Automation and energy-efficient equipment investments should improve operational reliability and lower production costs.

##### By-Product Utilization

Every year, the rose oil and jam industries produce substantial quantities of pomace and discarded petals. Preliminary investigations into the valorization of *Rosa damascena* by-products have confirmed the feasibility of extracting pectic polysaccharides with potential applications as food thickeners [22]. Nonetheless, rigorous cost–benefit assessments are required to substantiate their widespread industrial adoption [22]. By-products of the rose industry, like pomace and petal remnants, can be valued as a means of reducing waste and conserving resources. The process of valuing rose petals, pomace, and distillation residues for polysaccharide recovery is a prime illustration of how the circular economy can be integrated to reduce waste streams and produce valuable bioactive ingredients [169]. The creation of strong secondary raw material supply networks and thorough cost–benefit evaluations are necessary for the economic viability of large-scale by-product usage. These methods convert secondary raw materials into useful ingredients for foods, cosmetics, and nutraceuticals, which is in line with industrial sustainability goals.

##### Regulatory Frameworks

The safety and effectiveness of products in cosmetics and nutraceuticals will be guaranteed by establishing quality control criteria for rose polysaccharides, such as bioactivity thresholds and monosaccharide composition (e.g., HPLC profiles) [162]. In order to facilitate market entry and customer acceptability, it is imperative that clear regulatory standards, including quality control parameters and safety criteria, be established for rose polysaccharide products. The commercialization of innovative substances derived from roses will be supported and standardized guidelines will be developed more quickly if industry players and regulatory bodies work together. To close these gaps, industry and academia must work together, utilizing breakthroughs such as DES recycling systems and continuous-flow ultrasonic extractors. Techno-economic analyses (TEAs) and Life Cycle Assessments (LCAs) ought to direct sustainable commercialization routes [69,170]. The adoption of techno-economic analysis (TEA) and LCA-based evaluations is crucial to assessing the environmental impact and cost-effectiveness of large-scale extraction, even beyond regulatory quality standards [171]. Incorporating these frameworks into business operations will direct sustainable marketing and guarantee adherence to clean-label and eco-label regulations in the food and cosmetic industries.

Overall, the future application of rose polysaccharides will be influenced by the combination of breakthrough extraction technologies, extended species discovery, and the development of sustainable, standardized industrial processes. In order to overcome present obstacles and fully realize the promise of rose-derived polysaccharides as high-value functional components, cooperation between academics, industry, and regulatory bodies backed by thorough techno-economic and environmental assessments will be essential.

## 6. Conclusions

The extraction of polysaccharides from different portions of the rose plant (*Rosa* spp.) offers a viable path toward a variety of uses in functional foods, cosmetics, and pharmaceuticals. By scavenging free radicals, chelating metal ions, and modifying oxidative stress pathways, these bioactive polysaccharides, which are mostly made up of pectin, cellulose, hemicelluloses, and arabinogalactan, display strong antioxidant potential. The method used greatly affects extraction efficiency; traditional methods like ethanol and hot water extraction are straightforward but frequently result in poorer yields and the possible destruction of bioactive components. Advanced techniques include enzyme-assisted, pressured liquid, ultrasound-assisted, microwave-assisted, and supercritical fluid extraction; on the other hand, these techniques enhance polysaccharide recovery while preserving structural integrity and bioactivity. Enhancing the yield, purity, and functional properties of these methods is critical to commercialization. Nonetheless, challenges around polysaccharide degradation, inherent variability in composition based on species, and the scalability of processes exist, despite advances in extraction methods. Sustainable extraction methods, for example, alternative extraction methods such as green solvents, deep eutectic solvents, and enzymatic treatments, may improve efficiency and provide environmental alternatives to traditional extraction methods. Future research should focus on modeling-based approaches such as response surface methodology (RSM) to optimize process parameters and on alternative methods such as nanotechnology approaches for the targeted delivery of bioactive polysaccharides. In addition, additional research into underutilized *Rosa* spp. may maximize the potential for the use of polysaccharides derived from roses to be used in medicines and nutraceuticals. If these challenges are met through collaboration and technology, this will lead to the large-scale use of polysaccharides from roses and the development of high-value, environmentally friendly bioproducts.

## Figures and Tables

**Table 2:** Traditional techniques used in the extraction of polysaccharides in various parts of rose plants.

Plant Part	Extraction Methods	Conditions	Solvent/ Reagent Used	Characteristics & Yield	References
Leaves	Ethanol/Methanol Extraction	RT 12–24 h	80% Ethanol/ Methanol	Selective precipitation of polysaccharides, removes small molecules	[40]
	Hot Water Extraction	60–100 °C 2–5 h	Distilled water	Moderate yield (5–15%), presence of pectin and hemicelluloses	[68,69]
Petals	Ethanol/Methanol Extraction	RT, overnight	70–95% Ethanol/ Methanol	Removes impurities, enhances polysaccharide purity	[40,70]
	Hot Water Extraction	80–100 °C, 1–4 h	Distilled water	High yield (>15%), neutral polysaccharides	[32,69]
Stems	Ethanol/Methanol Extraction	RT, overnight	80% Ethanol/ Methanol	Purifies polysaccharides, removes phenolic compounds	[71,72]
	Hot Water Extraction	90 °C, 3–5 h	Distilled water	Moderate yield (5–15%), heterogeneous polysaccharide composition	[73,74]
Roots	Ethanol/Methanol Extraction	RT, 12–24 h	70–95% Ethanol/ Methanol	Precipitates polysaccharides, enhances purity	[12,75]
	Hot Water Extraction	90 °C, 2–4 h	Distilled water	Higher extraction efficiency, acidic polysaccharides	[76]

**Table 3:** Comparison of extraction methods for polysaccharides.

Extraction Method	Principle/Process	Key Parameters	Advantages	Limitations	References
Hot Water Extraction (HWE)	Heating plant material in water to dissolve water-soluble polysaccharides	60–100 °C, 1–4 h, water as solvent	Simple, cost-effective, environmentally friendly, preserves bioactivity, scalable	Long extraction time, risk of hydrolysis, low yield for some polysaccharides, co-extraction of impurities	[58,59,69,109,110,111,112]
Ethanol/Methanol Extraction (Precipitation)	Alcohol is added to aqueous extract to precipitate polysaccharides	70–90% (*v*/*v*) ethanol/methanol	Efficient for purification after aqueous extraction; removes proteins/phenolics	Not a primary extraction method; may not remove all impurities	[32,113]
Enzyme-Assisted Extraction (EAE)	Cell wall-degrading enzymes (e.g., cellulase, pectinase) break down plant matrix, releasing polysaccharides	Enzyme type/concentration, temperature (30–55 °C), pH, time	Higher yield, milder conditions, preserves structure, selective extraction	Cost of enzymes, risk of enzyme residue, optimization required	[112,113]
Ultrasound-Assisted Extraction (UAE)	Ultrasound waves disrupt cell walls, enhancing solvent penetration and mass transfer	20–60 kHz, 30–90 °C, 10–60 min	Shorter extraction time, increased yield, energy efficient	Possible degradation of polysaccharides at high power, equipment cost	[58,59]
Microwave-Assisted Extraction (MAE)	Microwaves rapidly heat plant material and solvent, causing cell rupture and release of polysaccharides	100–800 W, 60–120 °C, 5–30 min	Rapid, high efficiency, reduced solvent use, good yield	Risk of overheating/degradation, equipment cost	[58,92,111]
Pressurized Liquid Extraction (PLE)	Uses high pressure and temperature to enhance solvent extraction efficiency	50–200 °C, 10–20 MPa, 10–60 min	High yield, efficient, reduced solvent use	Specialized equipment, possible degradation at high temp	[110,114]
Supercritical Fluid Extraction (SFE)	Supercritical CO_2_ (often with co-solvents) extracts bioactive components under high pressure and moderate temperature	31–80 °C, 10–35 MPa, CO_2_/co-solvent	Selective, solvent-free product, preserves structure	High equipment cost, not suitable for all polysaccharides	[110]
Integrated/Hybrid Extraction	Combines two or more techniques to leverage synergistic effects (e.g., cavitation-enhanced enzyme penetration; rapid microwave heating with green DES)	Method-dependent; typically milder conditions, reduced enzyme dosage, shorter processing times	Higher yield and selectivity; improved antioxidant retention; lower energy/solvent use; greener	Optimization complexity; equipment integration and scale-up considerations	[106,107]

**Table 4:** Comparative performance of major extraction methods for polysaccharides from Rose plants.

Extraction Method	Yield (%)	Energy/Temp (°C)	Solvent-to-Solid Ratio (mL/g)	Extraction Time	Bioactivity Retention	References
Hot Water Extraction (HWE)	3.2–8.5%	80–100 °C continuous heating (high energy demand)	20–40:1	2–5 h	Moderate; risk of partial hydrolysis	[33,57]
Ethanol/Methanol Precipitation	(used for purification, not primary yield)	RT large alcohol volumes (high solvent use)	70–90% ethanol	12–24 h	High purity, but not efficient for yield	[32,64]
Enzyme-Assisted Extraction (EAE)	4.3–4.8%	45–60 °C mild heating; enzyme dose 1–3% (low energy)	15–20:1	80–120 min	High; mild conditions preserve activity	[32,95]
Ultrasound-Assisted Extraction (UAE)	6.5–15%	50–90 °C; 200–500 W	16–30:1	25–85 min	High; preserves antioxidant activity	[33,83,125]
Microwave-Assisted Extraction (MAE)	18–36%	60–120 °C; 400–600 W	12–15:1	5–20 min	High; fast heating avoids degradation	[126,127]
Pressurized Liquid Extraction (PLE)	15–30%	100–150 °C; 10–20 MPa	10–15:1	20–60 min	High; preserves structure	[77,79]
Supercritical CO_2_ Extraction (SFE)	10–25%	31–80 °C; 10–35 MPa	Low (CO_2_, with co-solvent 5–15%)	1–3 h	Very High; solvent-free extracts	[101,128]
DES-based Microwave/UAE (Emerging)	15–40% (varies)	60–90 °C 200–400 W	15–20:1	30–90 min	High; greener solvents, tunable	[90]

**Table 5:** Challenges in extraction and corresponding optimization strategies for Rose polysaccharides.

Challenges	Optimization Strategies	References
Low yield from woody tissues (Stem, roots) due to rigid cell walls composed of cellulose, hemicelluloses, and lignin.	Use enzymatic hydrolysis and mild acid or alkaline pretreatments to break down rigid cell walls, improving solubilization while preserving polysaccharide structure.	[60,129,130,132,140]
Polysaccharide degradation caused by high temperature, long extraction times, and harsh solvents.	Employ green extraction techniques such as ultrasound-assisted extraction (UAE) and microwave-assisted extraction (MAE) to reduce extraction time and temperature, minimizing degradation.	[10,22,134,135]
Variability in polysaccharide composition and antioxidant activity among rose species and plant parts due to genetic and environmental factors.	Standardize plant material selection, harvesting times, and preprocessing; develop species- and tissue-specific extraction protocols to ensure consistency.	[32,69,136,137,138]
Limited solubility of some polysaccharides in water, reducing extraction efficiency.	Use mixed solvents (e.g., aqueous ethanol, methanol) or deep eutectic solvents (DES) to improve solubility and maintain bioactivity.	[141,142,143,144]
Environmental concerns and high solvent consumption in traditional extraction methods.	Adopt sustainable and green extraction technologies such as supercritical fluid extraction (SFE), subcritical water extraction (SWE), UAE, and MAE to reduce solvent use and environmental impact.	[101,135,145,146]
Complex interactions of multiple extraction parameters make optimization challenging.	Apply statistical optimization methods like response surface methodology (RSM) to systematically optimize extraction conditions (temperature, time, solvent ratio, enzyme concentration).	[117,147,148]
Poor solvent penetration due to plant tissue structure limiting polysaccharide accessibility.	Use mechanical pretreatments such as grinding and ultrafine milling, combined with chemical pretreatments (alkaline or mild acid hydrolysis), to increase surface area and weaken cell walls.	[61,140]

## Data Availability

The original contributions presented in this study are included in the article. Further inquiries can be directed to the corresponding authors.
